# Exploring biologically-based complementary and alternative medicine use among Irish cancer survivors: findings from a national survey

**DOI:** 10.1093/oncolo/oyag127

**Published:** 2026-04-29

**Authors:** Clodagh Scannell, Erin Stella Sullivan, Karen Matvienko-Sikar, David Robert Grimes, Derek Power, Aoife Ryan

**Affiliations:** School of Food and Nutritional Sciences, College of Science, Engineering and Food Science, University College Cork, Cork, Ireland; Cancer Research @UCC, University College Cork, Cork, Ireland; Department of Nutritional Sciences, School of Life Course & Population Sciences, Faculty of Life Sciences & Medicine, King’s College London, London, United Kingdom; School of Public Health, University College Cork, Cork, Ireland; TCD Biostatistics Unit, Discipline of Public Health and Primary Care, School of Medicine, Trinity College Dublin, Dublin, Ireland; Cancer Research @UCC, University College Cork, Cork, Ireland; Department of Medical Oncology, Mercy University Hospital, Cork, Ireland; School of Food and Nutritional Sciences, College of Science, Engineering and Food Science, University College Cork, Cork, Ireland; Cancer Research @UCC, University College Cork, Cork, Ireland

**Keywords:** cancer, nutrition, dietary supplements, diets, survivorship, behaviours

## Abstract

**Background:**

Biologically based complementary and alternative medicine (BBCAM) includes special diets, dietary supplement and herbal remedies, not prescribed by a doctor or dietitian. The use of BBCAM is common among cancer survivors. BBCAM can interact with conventional treatments and unregulated products may cause harm. This study aimed to determine the prevalence, types, and motivations for BBCAM use among cancer survivors.

**Methods:**

A survey assessed clinical characteristics and BBCAM use in participants >18 yrs who had received cancer treatment in Ireland from 2018 to 2022.

**Results:**

Amongst 295 respondents (77% female, mean age 53 yrs), BBCAM use increased from 28% pre-diagnosis to 34% post-diagnosis (*p < *0.001). For BBCAM users (*n = *97, 33%), “daily-use” increased from 38% to 72% (*p < *0.001) post-diagnosis. Common types included: mineral/vitamin supplements (84%), dietary supplements (e.g. turmeric, coenzyme-Q10) (78%), herbal remedies/botanicals (e.g. mistletoe, St. John’s Wort, echinacea, ginseng) (50%), cannabis (21%), and other natural products (laetrile, shark cartilage, apricot kernels) (19%). Biological medicines (GcMAF, immuno-augmentative therapy) were used by 12% of BBCAM users. Special diets including dairy free (32%), gluten-free (19%), intermittent fasting (17%), ketogenic diet (15%), juicing/detox (10%) were also common. Perceived benefits included: improved well-being (63%) and reduced psychological stress (59%).

**Conclusion:**

BBCAM use increases after a cancer diagnosis. Patient perceived benefits highlight potential gaps in the current healthcare model, indicating a need for greater emphasis on safe survivorship care.

Implications for practice The use of biologically-based complementary and alternative medicine (BBCAM) increases significantly post cancer-diagnosis. Common practices include supplements, herbal remedies and restrictive diets. Despite the potential for interactions with conventional treatment, and the safety risks with unregulated products, cancer survivors report that BBCAM improves overall well-being and reduces psychological stress. It is imperative that healthcare professionals are better informed about the motivations for using BBCAM and be better able to provide evidence-based guidance for safer symptom management. As the staffing levels of dietitians are critically low, the development of evidence-based resources should be a priority.

## Introduction

In 2024, the World Health Organization (WHO) projected 35 million new cancer cases globally by 2050.^1^ Advances in screening and treatment have transformed cancer into a chronic condition, with people living longer post cancer-diagnosis.^2^^,^^3^ As a result, survivors now experience significant treatment and disease-related side-effects, such as fatigue, fear of recurrence, pain, psychological distress, and weight issues.^4^ This has led to a mounting interest in the long-term effects of cancer treatment.^4^ Alongside this, there has been an increase in the use of complementary and alternative medicine (CAM) among cancer survivors, with global prevalence rates ranging from 40%–87%.^5^^,^^6^ The National Centre for Complementary and Integrative Health (NCCIH) defines CAM as “*a group of diverse medical and health care systems, practices and products whose origins come from outside of mainstream medicine”.*^7^ Biologically based complementary and alternative medicine (BBCAM) includes special diets, dietary supplement and herbal remedies, not prescribed by a doctor or dietitian.^7^ BBCAM also includes unregulated forms such as cannabis-derived products, Gc protein-derived macrophage activating factor (GcMAF) and laetrile (Vitamin B17).

The use of BBCAM is not without risk and may cause harm through both direct and indirect mechanisms. Direct toxicity has been reported with certain agents, most notably laetrile (Vitamin B17), which can cause cyanide poisoning and severe adverse effects.^8^ Indirect toxicity may arise through pharmacokinetic interactions between herbal products and systemic anti-cancer therapies, particularly via modulation of cytochrome P450 enzymes and drug transporters, potentially leading to reduced treatment efficacy or increased toxicity.^9^ For example, agents such as St. John’s wort and grapefruit-derived compounds can significantly alter drug metabolism, raising concerns regarding subtherapeutic exposure or heightened toxicity.^10^ In addition, restrictive or unproven dietary practices may exacerbate malnutrition and cancer-related cachexia, contributing to clinically significant weight loss that necessitates procedural (e.g., enteral feeding tube placement) or medical (e.g., hospital admission) interventions, which in turn may delay or interrupt planned oncological treatment.^11^ Beyond physical harms, these approaches may also impose substantial financial toxicity, as patients often incur out-of-pocket costs for therapies that lack robust evidence of efficacy, further compounding the overall burden of cancer care.^12^ Despite the lack of consistent evidence to support the use of BBCAM, data from the National Health Interview Survey in the United States found that adult cancer survivors spent an annual estimate of $4 billion on vitamin/mineral supplements and $1.2 billion on herbal/non-vitamin supplements.^12^

From a safety and efficacy perspective, BBCAM differs greatly from other domains of CAM. For example, mind–body interventions (e.g., meditation, yoga) are generally supported by moderate evidence for improving patient-reported outcomes such as quality of life and distress, and are considered low risk when appropriately delivered.^13^^,^^14^ Conversely, a retrospective study using the National Cancer Database in the United States (*n = *190,1815) found that in patients with curable cancers, those who engage with CAM were more likely to refuse an element of conventional cancer treatment (CCT) and had a two-fold greater risk of death compared to those who did not use CAM (HR, 2.08; 95% CI, 1.50–2.90).^15^ Despite negative consequences, BBCAM is considered to have the highest-levels of use among cancer survivors when compared to other CAM modalities, as information is readily available through the media and social media.^16^ Additionally, the decision to commence CAM may be driven by information-seeking as a coping strategy, giving cancer survivors a perceived sense of control over their disease.^17^^,^^18^

In Ireland, oncology dietetic services are under-staffed, with just one dietitian for every 4500 cancer survivors.^19^ In the United States, 90% of oncology care is provided in outpatient settings, yet a national survey conducted in 215 outpatient cancer centres found that the ratio was 1 dietitian for every 2308 patients.^20^ This stark gap in service provision leads to information gaps that are often filled by unqualified and unreliable sources such as the internet and complementary therapists who promote CAM.^16^ Social media only amplifies the issue, with wellness influencers spreading unproven claims about cancer cures to vast audiences.^21^ Nutrition impact symptoms like fatigue and nausea, coupled with the high prevalence of multi-morbidity among survivors, underscores the need for reliable dietary guidance.^19^^,^^22^^,^^23^ Survivors are highly motivated to seek out health information, but gaps in formalised support during and after treatment drives them towards BBCAM as a perceived coping mechanism.^19^ Often the dietary changes that occur after self-direct research are unsuitable for patients on active treatment and may increase the risk of developing cancer-associated malnutrition.^19^^,^^24^

Irish research on BBCAM is limited, with existing studies focusing on specific groups, such as breast cancer^25^^,^^26^ or paediatric populations.^27^^,^^28^ In addition, many of these studies are >10 years old and likely not representative of CAM use in Ireland today. A more recent Irish national survey briefly examined the use of BBCAM in cancer survivors but was not the study’s main objective.^19^ Given the growing rates of interest in BBCAM and potential implications for health, it is necessary to establish trends and drivers of interest.^19^^,^^29^ The aim of this study was to determine the prevalence and types of BBCAM being used by Irish cancer survivors. This study is the first of its kind to examine beliefs surrounding the safety, efficacy, mechanism, benefits and consequences of BBCAM in an Irish context.

## Methods

### Survey design

A national cross-sectional survey was conducted using a newly designed 65-question questionnaire (Supplementary Material). Participants were asked about demographics, clinical characteristics and details of their cancer diagnosis and treatments (past, present, future treatments). Participants were also asked about their own personal use of CAM before/after cancer-diagnosis, according to the 5 domains set out by the NCCIH.^8^ These include Whole Medical Systems (e.g., homeopathy, traditional Chinese medicine), Mind-Body Medicine (e.g., meditation, mindfulness), Manipulative and Body Based Practices (e.g., massage, reflexology), Energy Medicine (e.g., Reiki, Tai Chi) and Biologically Based Forms (e.g., self-prescribed dietary supplements or special diets). The survey explored all types of CAM as described above, with the focus of this analysis being BBCAM.

Participants were asked to respond to a number of statements using 5-point Likert scales. Specifically, these explored perceptions around the safety, efficacy, and the mechanisms of CAM. Checkboxes for multiple responses were utilised for information sources, perceived positive/negative effects of CAM, barriers to using CAM, and where participants accessed CAM.

To address inconsistencies in CAM research, this study followed Horneber et al. (2012) reporting standards.^5^ To ensure a standardised interpretation, a definition and examples of CAM were provided at the beginning of the survey. Participants could re-visit this section at any point to receive reminders. The survey utilized Likert scales, radio buttons, and checkboxes, with some open-text fields. To ensure accessibility, patient representatives from Irish cancer charities reviewed the survey for readability and acceptability.

### Study population

Participants were eligible if they were aged >18 years and diagnosed/treated for cancer in Ireland in the last 5 years. Recruitment involved advertising in clinical settings, through cancer charities, and on social media. Initially intended for in-person dissemination, the survey was launched online due to Covid-19 restrictions. Advertisements and personal letters were sent to healthcare professionals (HCPs), cancer support charities and cancer survivors (*n = *1000) to encourage participation.

### Statistical analysis

Statistical analysis was performed using SPSS Statistics for Windows v28.0.^30^ Most data were categorical and analysed using Chi-square tests with Bonferroni adjustments. Continuous variables (e.g. age) were analysed with independent t-tests. Missing data were considered missing at random and not replaced.

## Results

### Demographics and clinical characteristics

There were 295 respondents, 77% were female. Mean age was 53 years (*SD* = 12 years). Table 1 shows the proportion of BBCAM users and non-users in relation to various demographic and clinical characteristics. Notably, BBCAM users were more likely than non-users to be female (87% vs. 13% *p = *0.007) and well educated, having obtained a Level 9 master’s degree/postgraduate diploma (26% vs. 19%, *p = *0.002). The most common cancer types were breast (52%), gynaecologic/genitourinary (11%) and gastrointestinal cancers (9%). Notably, BBCAM users were more likely than non-users to have breast cancer (59% vs. 49%, *p = *0.043) and have stage 1 disease (22% vs. 8%, *p = *0.039). Patients diagnosed with gastrointestinal cancers were less likely to use BBCAM (4% vs. 11%, *p = *0.045). Additionally, patients with a more recent cancer diagnosis (within the last year) were less likely to use BBCAM (*p = *0.046). Although not statistically significant, BBCAM users (21%) were less likely than non-users (29%) to have active disease.

**Table 1.** Demographics and clinical characteristics stratified by BBCAM use post cancer diagnosis.

	BBCAM use (*n = *97)	No BBCAM use (*n = *198)	Total (*n = *295)	*p*-Value
**Gender**				*0.007*
Male	13 (13.4%)	55 (27.7%)	68 (23.1%)	
Female	84 (86.6%)	143 (72.3%)	227 (76.9%)	
**Mean age (SD)**	51.5 (10.9)	53.3 (12.1)	52.7 (11.8)	0.470
**Age Categories**				
<35 years	8 (8.2%)	13 (6.6%)	21 (7.1%)	NS
36–50 years	41 (42.3%)	73 (37.1%)	114 (38.6%)	NS
51–64 years	36 (37.1%)	73 (37.1%)	110 (37.4%)	NS
65+ years	12 (12.4%)	38 (19.3%)	50 (16.9%)	NS
Cancer Site	*n = *96	*n = *195	*n = *291	0.112
Breast	57 (59.4%)	95 (48.7%)	152 (52.2%)	*0.043*
Gynae/GU*	12 (12.5%)	20 (10.3%)	32 (11%)	NS
Gastrointestinal	4 (4.2%)	22 (11.3%)	26 (8.9%)	*0.045*
Haematological	5 (5.2%)	12 (6.2%)	17 (5.8%)	NS
Lung	3 (3.1%)	13 (6.7%)	16 (5.5%)	NS
Prostate	5 (5.2%)	5 (2.6%)	10 (3.4%)	NS
Head and Neck	–	7 (3.6%)	7 (2.4%)	NS
Other	10 (10.4%)	21 (10.8%)	31 (10.7%)	NS
**Disease Status**	*n = *90	*n = *191	*n = *281	0.490
Active Disease	19 (21.1%)	56 (29.3%)	75 (26.7%)	NS
Recurrence	5 (5.6%)	15 (7.9%)	20 (7.1%)	NS
Cured/Remission	65 (72.3%)	119 (62.3%)	184 (65.5%)	NS
Other/Don’t Know	1 (1.1%)	1 (0.5%)	2 (0.7%)	NS
**Cancer Stage**	*n = *93	*n = *190	*n = *283	0.200
0/in situ	4 (4.3%)	12 (6.3%)	16 (5.7%)	NS
Stage 1	20 (21.5%)	23 (12.1%)	43 (15.2%)	*0.039*
Stage 2	23 (24.7%)	45 (24.3%)	70 (24.8%)	NS
Stage 3	25 (26.9%)	50 (26.3%)	73 (25.8%)	NS
Stage 4	19 (20.4%)	48 (25.3%)	67 (23.7%)	NS
Don’t Know	2 (2.2%)	12 (6.3%)	14 (4.9%)	NS
**Diagnosis Time Frame**	*n = *90	*n = *190	*n = *280	
In the last year	14 (15.6%)	46 (24.2%)	60 (21.4%)	*0.046*
Over a year ago	76 (84.4%)	144 (75.8%)	220 (78.6%)	NS

* Gynaecologic/genitourinary, NS: not significant.

### Cancer treatment patterns

When asked about previous cancer treatments, 72% of the total study cohort had undergone surgery, 70% had received chemotherapy, 58% radiotherapy, 36% hormone therapy, 14% immunotherapy and 14% targeted therapy. In relation to past treatments, BBCAM users were more likely to have undergone surgery than non-users (80% vs. 69%, *p = *0.037). There were no significant differences between groups in relation to other past treatments (e.g., chemotherapy, radiotherapy, hormone therapy, immunotherapy). At the time of survey completion, it was estimated that 38% of the total study cohort were not undergoing any form of active treatment, 28.5% were receiving hormone therapy, 15% were receiving chemotherapy, 11% were receiving targeted therapy and 5% were receiving immunotherapy. BBCAM users were less likely than non-users to be currently receiving chemotherapy (8% vs. 19%, *p = *0.015) and targeted therapy (6.6% vs. 14%, *p = *0.028). In relation to future treatments, 47% of the total study cohort had no plans for any future treatment, 22% had plans for hormone therapy, 10.5% chemotherapy, 9.2% targeted therapy and 7.5% radiotherapy. BBCAM users (6%) were less likely than non-users (13%) to have plans for future chemotherapy (*p = *0.019).

### Types of BBCAM used

Among participants reporting use of BBCAM following a cancer diagnosis (*n = *97), the most used modalities were vitamin and mineral supplements (83.5%), dietary or food supplements (e.g., garlic, ginger, turmeric/curcumin, co-enzyme Q10) (78.4%), and herbal remedies/botanicals (49.5%). Notably, when asked about unregulated products, 21% of BBCAM users (*n = *97) reported using cannabis-derived tetrahydrocannabinol (THC) and 7% reported using laetrile (Vitamin B17). The use of biological medicines, several of which are banned/unapproved in the US and Europe (e.g. GcMAF, antineoplastics, 714S, Immuno-augmentative therapy, melatonin) were used by 12.4%. When BBCAM users (*n = *97) were asked about special diets, 32% reported following a dairy-free diet, 18% had tried a gluten-free diet (not indicated due to coeliac disease) and 17% had tried intermittent fasting. Additional details about commonly used BBCAM and special diets can be found in Table 2.

**Table 2.** Types of BBCAM reported by BBCAM users post cancer diagnosis (*n = *97).

BBCAM types	BBCAM users	Total (%)
	*n = *97	*n = *295
Vitamin/Mineral Supplement	81 (83.5%)	27.5%
Dietary Supplement*	76 (78.4%)	25.7%
Herbal Remedies/Botanicals**	48 (49.5%)	16.3%
Cannabis THC	20 (20.6%)	6.8%
Natural Products***	18 (18.6%)	6.1%
Biological Medicines****	12 (12.4%)	4.1%
Quercetin	11 (11.3%)	3.7%
Colloidal Silver	9 (9.3%)	3.1%
Laetrile/Vitamin B17	7 (7.2%)	2.4%
Berberine	6 (6.2%)	2.0%
IV Vitamin C/Ascorbic Acid	6 (6.2%)	2.0%
Sodium Bicarbonate	5 (5.2%)	1.7%
***Types of Special Diets***		
Dairy Free Diet	31 (32%)	10.5%
Gluten Free (If not coeliac)	18 (18.6%)	6.1%
Intermittent Fasting	16 (16.5%)	5.4%
Ketogenic Diet (Low Carb, High Fat)	15 (15.5%)	5.1%
Green Juicing Diet	10 (10.3%)	3.4%
Detoxification Diet	10 (10.3%)	3.4%
Alkaline Diet	7 (7.2%)	2.4%

* Examples include; Garlic, Ginger, Turmeric/Curcumin, Coenzyme Q10,

** examples include; Mistletoe, St. John’s Wort, Echinacea, Ginseng, Ginkgo Biloba, Elderberry, Valerian, Chamomile,

*** examples include; Shark Cartilage, Laetrile/Vitamin B17/Apricot Kernels,

**** examples include; GcMAF, Antineoplastics, 714X, Immuno-augmentative therapy, Melatonin.

### Patterns of BBCAM use

Patterns of BBCAM use before and after cancer diagnosis are presented in Table 3. In total, 69% (*n = *204) reported having ever used BBCAM, with the use of BBCAM increasing significantly from 28.1% pre-diagnosis to 33.5% to post-diagnosis (*p < *0.001). Notably, “daily-use” of BBCAM for BBCAM users (*n = *97) increased from 38% pre-diagnosis to 72% post-diagnosis (*p < *0.001). Interestingly, 26% who reported not using BBCAM prior to diagnosis, commenced using BBCAM post-diagnosis (*p < *0.001). A small percentage (2%) of BBCAM users reported using BBCAM as an alternative to conventional cancer treatment.

**Table 3.** Prevalence of BBCAM use pre and post cancer diagnosis.

	Daily		Weekly		Monthly		Yearly		Never	
	*Pre*	*Post*	*Pre*	*Post*	*Pre*	*Post*	*Pre*	*Post*	*Pre*	*Post*
***BBCAM users (*n* = 97)***	36 (37.9%)	70 (72.2%)	10 (10.5%)	8 (8.2%)	13 (13.4%)	16 (16.5%)	11 (11.6%)	3 (3.1%)	25 (26.3%)	0
***p-*Value**	*p < *0.001		*p = *0.307		*p = *0.260		*p < *0.001		*p < *0.001	
***Non-users post diagnosis (*n* = 192)***	4 (2.1%)	–	–	–	3 (1.6%)	–	4 (2.1%)	–	181 (94.3%)	192 (100%)

*p*-Value depicting comparison between BBCAM users (*n* = 97) frequency of use before and after cancer-diagnosis.

### Symptoms experienced

Common symptoms experienced by the total cohort were fatigue (57%), disturbed sleep (49%) and fear of recurrence (42%). BBCAM users (*n = *97) were more likely to experience fatigue (59.6% vs. 50.5%, *p = *0.049) and fear of recurrence (46.4% vs. 39.9%, *p = *0.038) than non-users. BBCAM users (0%) were less likely than non-users (8%) to report bowel incontinence (*p = *0.004).

### Perceptions of BBCAM

Perceived positive effects of BBCAM reported by the entire cohort (*n = *295) were to improve overall well-being (53%), reduce psychological stress (47%) and improve quality of life (42%). Notably, BBCAM users were more likely than non-users to report that BBCAM would “slow tumour growth” (26% vs. 11%, *p = 0.011*) and “kill the tumour” (9% vs. 2.5%, *p = 0.010*). Interactions with conventional treatment (34%), delaying conventional treatment (32.5%) and refusing conventional treatment (31.5%) were deemed potential negative effects of BBCAM by the total study cohort (*n = *295). BBCAM users (*n = *97) were more likely to than non-users to report that BBCAM use could cause tension with healthcare professionals (41% vs. 23%, *p* < 0.001) and cause financial stress (37% vs. 12%, *p = *0.002). Additional details about the positive and negative effects of CAM can be found in Table 4.

**Table 4.** Perceived positive and negative effects of BBCAM stratified by BBCAM use post diagnosis.

Perceived positive effects of CAM	BBCAM use	No BBCAM use	Total	*p*-Value
	*n = *97	*n = *198	*n = *295	
Improve overall wellbeing	61 (62.9%)	96 (48.5%)	157 (53.2%)	*0.020*
Reduce psychological stress	57 (58.8%)	81 (40.9%)	138 (46.8%)	*0.004*
Improve quality of life	53 (54.6%)	71 (35.9%)	124 (42%)	*0.002*
Lessen the side effects of conventional treatment	54 (55.7%)	66 (33.3%)	120 (40.7%)	*<0.001*
Provide a support network for cancer survivors	33 (34%)	51 (28.5%)	84 (28.5%)	NS
More open to conventional treatment	33 (34%)	38 (19.2%)	71 (24.1%)	*0.005*
Optimise the effects of conventional treatment	40 (41.2%)	33 (16.7%)	73 (24.7%)	*<0.001*
Lessen symptoms of cancer	26 (26.8%)	39 (19.7%)	65 (22%)	NS
Slow tumour growth	25 (25.8%)	22 (11.1%)	47 (15.9%)	*<0.001*
Improve social relationships for the cancer survivor	23 (23.7%)	24 (12.1%)	47 (15.9%)	*0.011*
No positive effects	1 (1.0%)	16 (8.1%)	17 (5.8%)	*0.015*
Kill the tumour	9 (9.3%)	5 (2.5%)	14 (4.7%)	*0.010*
Improve the HCP/patient relationship	3 (3.1%)	6 (3.0%)	9 (3.1%)	NS
Reduce financial stress	5 (5.2%)	3 (1.5%)	8 (2.7%)	NS
All of the above	3 (3.1%)	4 (2.0%)	7 (2.4%)	NS

Perceived Negative Effects of CAM	BBCAM use	No BBCAM	Total	*p*-Value
	*n* = 97	*n* = 198	*n* = 295	
Interact with conventional Tx	39 (40.2%)	61 (30.8%)	100 (33.9%)	NS
Can cause cancer survivors to delay conventional Tx	31 (32%)	65 (32.8%)	96 (32.5%)	NS
Can cause cancer survivors to refuse conventional Tx	32 (33%)	61 (30.8%)	93 (31.5%)	NS
Cause its own side effects	31 (32%)	59 (29.8%)	90 (30.5%)	NS
Can cause tension with HCPs	40 (41.2%)	45 (22.7%)	85 (28.8%)	*<0.001*
Cause financial stress	36 (37.1%)	40 (20.2%)	76 (25.8%)	*0.002*
Cause obsessive or restrictive behaviours	16 (16.5%)	28 (14.1%)	44 (14.9%)	NS
No negative effects	10 (10.3%)	26 (13.1%)	36 (12.2%)	NS
Worsen side effects of conventional treatment	13 (13.4%)	26 (13.1%)	39 (13.2%)	NS
Worsen symptoms of cancer	9 (9.3%)	26 (13.1%)	35 (11.9%)	NS
Worsen overall well-being	11 (11.3%)	21 (10.6%)	32 (10.8%)	NS
Cause unnecessary psychological stress	12 (12.4%)	20 (10.1%)	32 (10.8%)	NS
Enhance tumour growth	8 (8.2%)	23 (11.6%)	31 (10.5%)	NS
Worsen quality of life	5 (5.2%)	18 (9.1%)	23 (7.8%)	NS
Can cause social problems	7 (7.2%)	11 (5.6%)	18 (6.1%)	NS
All of the above	3 (3.1%)	14 (7.1%)	17 (5.8%)	NS

NS: not significant.

**Figure 1.**
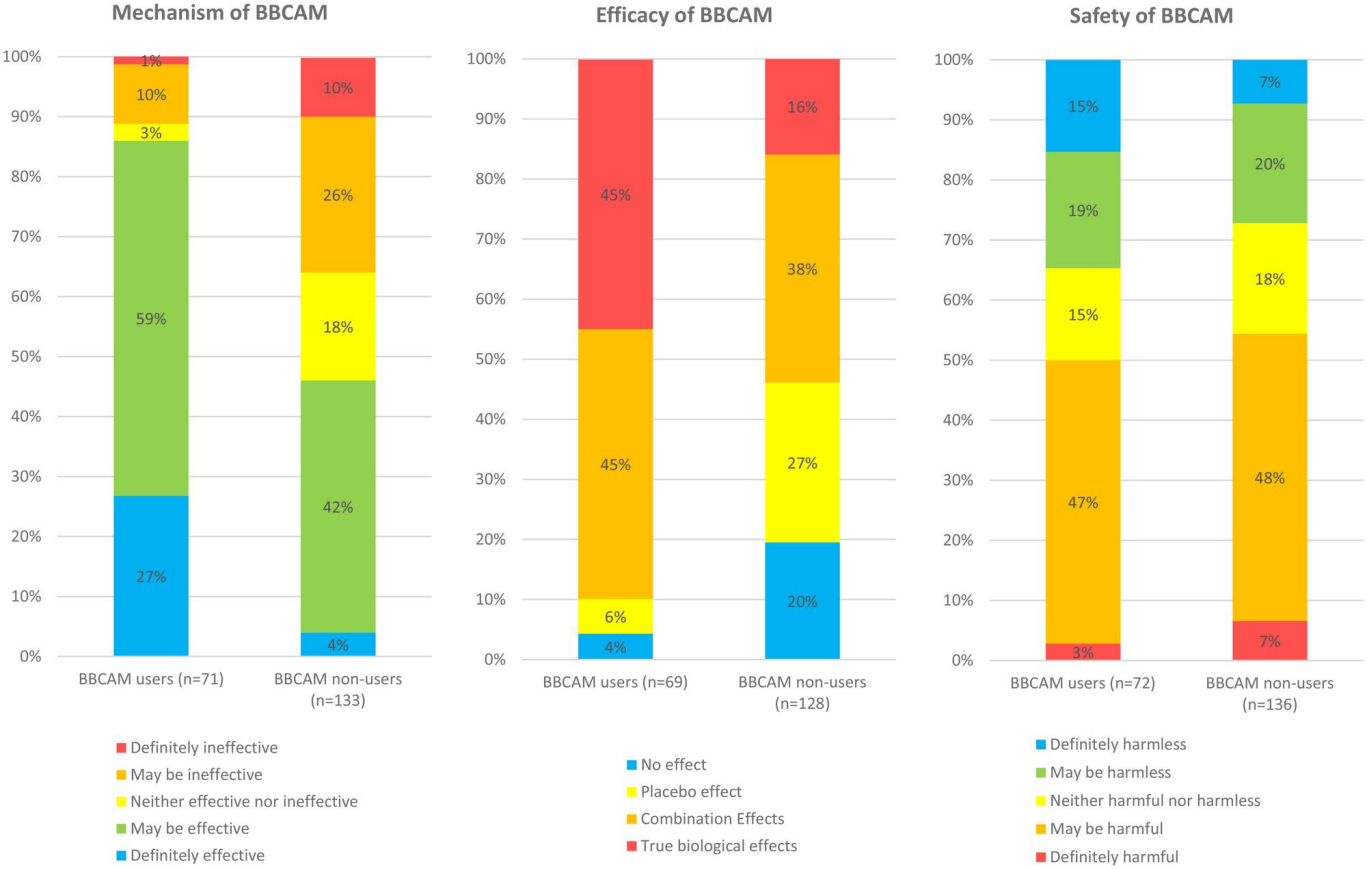
Mechanism, Safety and Efficacy of BBCAM as perceived by BBCAM users (*n = *97) and BBCAM (*n = *198).

When asked about the mechanism of BBCAM, BBCAM users were more likely than non-users to think that BBCAM has true biological effects (45% vs. 16%, *p* < 0.001). An additional 45% of BBCAM users were of the belief that BBCAM has a combination of true biological effects and a placebo effect. Amongst non-users, 19% believe BBCAM has no effect. When asked about the efficacy of BBCAM, BBCAM users were more likely than non-users to report that BBCAM was *“definitely effective”* (27% vs. 4%, *p* < 0.001). Conversely, BBCAM users were less likely than non-users to report that BBCAM was “definitely ineffective” (1.4% vs. 9.8%, *p* = 0.008). When asked about the safety of BBCAM, only a small percentage of BBCAM users (3%) considered BBCAM to be “definitely harmful”. BBCAM users (15.3%) were more likely than non-users to say that BBCAM was “definitely harmless” (15.3% vs. 7.4%, *p = *0.014). See Figure 1.

### Sources of information and accessing CAM

The most reported sources of information were Google (39%), cancer support centres (32%) and friends (32%) (See Table 5). BBCAM users (52%) were significantly more likely than non-users (33.3%) to report using Google to find out information about CAM (*p = *0.003). BBCAM users were more likely than non-users to have obtained information about CAM from complementary therapists (41% vs. 12%, *p < *0.001), scientific literature (39% vs. 15%, *p < 0.001*) and books (32% vs. 8%, *p* < 0.001). The total study cohort (*n = *295) reported accessing CAM from private CAM therapists (35%) (e.g. herbalist, naturopath), health food shops (31%) and cancer support centres (26%) (See Table 6). BBCAM users were more likely than non-users to have accessed CAM from private CAM therapists (58% vs. 23%, *p < 0.001*), health food shops (55% vs. 19%, *p < 0.001*) and buying products online (16.5% vs. 3.5%, *p < 0.001*). These figures likely indicate where non-users post diagnosis accessed CAM prior to being diagnosed with cancer or include other CAM modalities (e.g. massage, yoga, meditation).

**Table 5.** Information sources about CAM stratified by BBCAM use post diagnosis.

Information sources	BBCAM	No BBCAM use*	Total	*p*-Value
	*n = *97	*n = *198	*n = *295	
Googling a Question	50 (51.5%)	66 (33.33%)	116 (39.3%)	*0.003*
Complementary Therapists	40 (41.2%)	23 (11.6%)	63 (21.4%)	*<0.001*
Scientific Literature	38 (39.2%)	30 (15.2%)	68 (23.1%)	*<0.001*
Cancer Support Centres	36 (37.1%)	58 (29.3%)	94 (31.9%)	0.176
Friends	36 (37.1%)	56 (28.3%)	92 (31.2%)	0.124
Books	31 (32%)	16 (8.1%)	47 (15.9%)	*<0.001*
Health Food Shops	30 (30.9%)	18 (9.1%)	48 (16.3%)	*<0.001*
Web Pages (blogs)	27 (27.8%)	25 (12.6%)	52 (17.6%)	*<0.001*
Family	26 (26.8%)	34 (17.2%)	60 (20.3%)	*0.053*
Other Patients	20 (20.6%)	31 (15.7%)	51 (17.3%)	0.290
Social Media	18 (18.6%)	30 (15.2%)	48 (16.3%)	0.457
Colleagues	15 (15.5%)	14 (7.1%)	29 (9.8%)	*0.023*
Podcasts	11 (11.3%)	17 (8.6%)	28 (9.5%)	0.448
Radio	11 (11.3%)	11 (5.6%)	22 (7.5%)	0.076
Newspapers	10 (10.3%)	20 (10.1%)	26 (8.8%)	0.956
Acquaintances	10 (10.3%)	14 (7.1%)	24 (8.1%)	0.339
TV	10 (10.3%)	13 (6.6%)	23 (7.8%)	0.260
Magazines	9 (9.3%)	17 (8.6%)	26 (8.8%)	0.844
Hospital Staff	7 (7.2%)	12 (6.1%)	19 (6.4%)	0.704
Gym	3 (3.1%)	1 (0.5%)	4 (1.4%)	0.071

* May include BBCAM non-users obtaining information about other CAM modalities (e.g. massage, reiki, acupuncture).

**Table 6.** Where CAM was accessed stratified by BBCAM use post diagnosis.

Where CAM is accessed	BBCAM	No BBCAM*	Total	*p*-Value
	*n = *97	*n = *198	*n = *295	
Private CAM Therapists**	56 (57.7%)	46 (23.2%)	102 (34.6%)	*<0.001*
Health Food Shops	53 (54.6%)	38 (19.2%)	91 (30.8%)	*<0.001*
Cancer Support Centres	30 (30.9%)	46 (23.3%)	76 (25.8%)	0.156
Online Stores	16 (16.5%)	7 (3.5%)	23 (7.8%)	*<0.001*
Other Patients	2 (2.1%)	4 (2.0%)	6 (2.0%)	0.981
Hospital	–	5 (2.5%)	5 (1.7%)	0.114
Gym	1 (1.0%)	1 (0.5%)	2 (0.7%)	0.605

* May include BBCAM non-users accessing other CAM modalities (e.g. massage, reiki, acupuncture).

** Examples include; herbalists, nutritionists, naturopaths, chiropractor and aromatherapist.

## Discussion

The aim of this study was to conduct the first national survey examining the prevalence and types of BBCAM being used by Irish cancer survivors. Findings revealed that 69% of respondents had previously used BBCAM in their lifetime, with use increasing significantly post-diagnosis. BBCAM was primarily used to improve well-being and alleviate psychological stress. However, a notable number of participants followed restrictive diets, such as dairy-free, ketogenic, or juicing regimens which can increase the risk of cancer-associated malnutrition. Furthermore, many reported using natural products and biological medicines, including unregulated or banned substances. There were high levels of variability in knowledge relating to the safety, efficacy and mechanism of BBCAM, which may stem from deceptive information found online or poor health literacy.

Results from this present study indicate that cancer survivors who have been diagnosed more recently were less likely to use BBCAM than those diagnosed >1 year ago. This is consistent with previous work from Wolf et al. (2022) who reported that time since diagnosis is a significant predictor of CAM-use.^31^ Notably, patients diagnosed with gastrointestinal cancers were less likely to use BBCAM (4% vs. 11%, *p = *0.045) which may suggest that these individuals were potentially experiencing symptoms associated with gastrointestinal disease and as such, were less likely to ingest BBCAM. This present study found that BBCAM users were significantly more likely to have stage 1 disease and have previously undergone surgery when compared to those who didn’t use BBCAM post-diagnosis. If we consider cancer survivors who have been diagnosed over a year ago and had early stages of disease, this likely indicates the unmet needs of cancer survivors who are diagnosed early, treated effectively with surgery, and quickly discharged without any regular oncology follow-up. This may contribute to the fear of recurrence and subsequent BBCAM use as reported in this present study. It has been previously acknowledged that there is a close link between CAM and anxiety. A British survey of 600 cancer patients found that those using CAM were more anxious than those just receiving conventional cancer treatment.^32^

Results from this present study found that BBCAM users report using BBCAM to improve overall wellbeing and reduce psychological stress. Oh et al. (2010) reported that 90% of Australian cancer patients (*n = *361) believed that BBCAM provided them with health benefits, with 56% reporting that it improved quality of life.^33^ Results from this present study suggests that when psychological needs are not met, cancer survivors turn to BBCAM to alleviate their anxiety and fear of recurrence. This may indicate that there are gaps in services available to address the psychological needs of cancer survivors, particularly those post-treatment, and an integrated approach is required.^28^ Additionally, a considerable number of participants (57%) in this present study reported experiencing fatigue, which may contribute to the increased consumption of supplements which are often marketed as a solution to improve energy levels. Recommendations from the American Society of Clinical Oncology (ASCO) includes data from 113 RCTs and found that there was no evidence to support the use of any dietary supplements (ginseng, mistletoe, coenzyme Q10) in the management of cancer-related fatigue.^34^

Consistent with other cross-sectional studies of oncology populations, our findings indicate that a noteworthy subset of Irish cancer survivors hold beliefs about BBCAM that suggest potential anticancer effects. In our cohort, 26% of participants reported that BBCAM use would *slow tumour growth* and 9% believed that it would *kill the tumour*, although we did not explicitly ask about recurrence-related motivations. Other cross-sectional data indicates that survivors report using dietary supplements explicitly to reduce the risk of recurrence. In a UK multi-centre survey of 1049 breast, prostate and colorectal cancer survivors, Conway et al. found that 19% believed supplements were important for lowering recurrence risk (and supplement users were three times more likely to hold this belief).^35^ However, these trends contrast sharply with current evidence-based guidance from the World Cancer Research Fund (WCFR) who do not recommend dietary supplement use for the prevention of cancer, nor for preventing recurrence.^36^ Together, these data underscore a persistent perception that BBCAM can influence cancer outcomes, reinforcing the need for clear counselling aligned with WCRF recommendations.^36^

Self-guided dietary changes including restricting or eliminating foods are common in cancer, and are often used by patients in an attempt to “cure” cancer or alleviate nutrition impact symptoms.^24^ In this present study a considerable number of participants report following a special diet, with one-third following a dairy-free diet. This is of particular concern as cancer survivors on active treatment likely require a high protein, high calorie diet.^11^ The European Society for Clinical Nutrition and Metabolism (ESPEN) do not recommend the use of restrictive or “fad” diets to prevent or treat cancer.^11^ In fact, animal proteins provide a greater anabolic stimuli and are considered better for muscle health.^37^ It has even been suggested that animal protein should make up >65% of total daily protein intake to support muscle health and avoid malnutrition.^37^ Cancer-associated malnutrition is associated with decreased tolerance to treatment, poorer quality of life and reduced overall survival.^38^

There are over 200 000 cancer survivors in Ireland, not including those prior to Irish registry records (1994—2020).^39^ In this present study, most cancer groups were represented in a proportion close to that of population prevalence, excluding breast and prostate cancer. Breast cancer is the most common female cancer type in Ireland (30% incidence between 2019-2021) and as a result, a high level of engagement with this study was expected.^39^ Irish research conducted by Fox et al. (2013) found that in a cohort of 406 cancer survivors, 56% of females diagnosed with breast cancer had used some form of CAM since diagnosis, adding that biologically-based forms were the most popular.^25^ It has been well established that females with breast cancer have higher information needs and are more likely to initiate BBCAM after diagnosis.^25^

This current study shows that there are high levels of variability in knowledge relating to the safety, efficacy and mechanism of BBCAM, which may stem from mis/disinformation found online or poor health literacy.^19^^,^^40^ Reports of the effectiveness of BBCAM usually stems from anecdotal evidence, misconceptions and biased opinions.^41^ A considerable number of BBCAM users in this present study reported using natural products and biological medicines which likely include unregulated and potentially illegal substances. In recent years, the internet and social media have become a valuable resource for cancer survivors but has now also become a gateway to easily accessible dangerous substances. An example of one of these potentially fatal substances is laetrile (or vitamin B17). A Cochrane review investigating the use of laetrile as a treatment for cancer found that there was no evidence to support its efficacy, and ingestion can result in cyanide poisoning.^8^ A systematic review of 23 randomised controlled trials evaluating the use of cannabis-based medicines for treatment of chemotherapy-induced nausea concluded that the trials were generally of low quality and that the benefit of cannabis-derived products over prescribed anti-nausea medications cannot be determined. Notably, participants who used cannabis-based products were more likely to experience debilitating side effects (e.g. dizziness, sedation) than those prescribed either the placebo or anti-nausea medication.^42^

This present study found that 84% of BBCAM users (*n = *97) reported using supplements. Similar studies have reported the use of vitamin and mineral supplements to be 19-81% in those diagnosed with cancer. Despite no evidence to show that supplements can impact cancer prognosis, cancer survivors often turn to antioxidant use with the misconception that they will help to dampen oxidative stress and improve the subsequent side effects that occurs concurrently with CCT.^16^ However, increasing oxidative stress is one mechanism by which cytotoxic therapies exert their effects, and it has been suggested that high-dose antioxidant supplementation may reduce the efficacy of CCT.^31^

The ATBC study is a landmark trial which examined the effect of vitamin E and beta carotene on the incidence of lung cancer in male smokers.^43^ Results from this trial suggests that high doses of beta-carotene (20 mg/day) may actually increase the incidence of lung cancer in male smokers compared to a placebo (57.2 vs. 47.7 incidence per 10 000 person/year).^43^ This study also found that mortality was 8% higher in those who received beta-carotene when compared to those who received the placebo (*p = 0.02*). Due to the strength of the evidence, the ATBC study had to be prematurely terminated and although published in 1994, still informs clinical practice today.^43^ In addition, folate metabolism is a target for various chemotherapy drugs (e.g. 5-fluorouracil), therefore controlled timing of folinic acid is required to prevent severe cytotoxic effects.^44^ A systematic review of 40 publications concluded that in studies focusing on antifolate therapies, lower folate/folic acid intake was associated with a higher risk of toxicities (12 out of 22 studies), but that in studies focusing on fluoropyrimidine treatments, higher folate intake were associated with a higher risk of toxicities (8 out of 14 studies).^44^ Therefore it could be suggested that self-prescribed, high-dose, folic acid supplements may place vulnerable patients at an even greater risk of toxicity.^44^ ESPEN does not recommend the use of high-dose micronutrients, and advise that they should only be supplied in amounts to meet the recommended daily allowance when there is a known deficiency.^11^ As previously discussed, the WCRF does not recommend the use of high-dose dietary supplements for cancer prevention.^36^ This is particularly relevant for cancer survivors in remission who are hoping to prevent disease recurrence.

The use of herbal remedies is also highly prevalent, with reported ranges of 26-63%.^45–47^ This present study shows 16.5% of the total cohort reported using herbal remedies/botanicals. It has been suggested that some products, St John’s wort for example, has the potential to reduce the effects of some anti-cancer treatments (e.g. irinotecan and docetaxel). This is of concern as St John’s wort is easily accessible and usage often not disclosed to healthcare professionals.^41^^,^^48^ Herbal remedies are not recommended by any reputable cancer organisation for the prevention or treatment of cancer, nor for the management of cancer-related symptoms.^11^

### Strengths and limitations

The length of the survey allowed for a detailed analysis of BBCAM use but may have reduced rates of completion. Recruitment challenges during the Covid-19 pandemic further limited the scope of the study, as in-person distribution was not feasible.^49^ For the entire duration of this study, oncology clinics were running primarily via telemedicine and as such, in-person advertisement was also not effective. As the survey was not as large, or the sample as representative as hoped, our estimate of the prevalence of BBCAM must be interpreted with caution. Despite specific efforts to recruit men, they are still under-represented in this study, with only 21% of respondents being male. In addition, given the self-reported nature of the survey, the findings are subject to potential recall bias, as participants may not have accurately remembered or reported their use of BBCAM.

This study also highlights the potential impact of pandemic-related misinformation and conspiracy theories on attitudes towards healthcare and BBCAM.^50^^,^^51^ This may have contributed to underreporting of illegal substances such as laetrile and GcMAF. Despite these limitations, the survey provides valuable insights into BBCAM use among Irish cancer survivors, reflecting international trends.

These results indicate the need for an integrated approach to address unmet needs. Disseminating evidence-based information can empower cancer survivors to make informed choices. In order to ensure the integrity of cancer care provided, the development of evidence-based guidelines should take priority.^52^ HCPs often hesitate to discuss CAM due to their own perceived lack of expertise and lack of guidance.^41^ Open dialogue can reduce reliance on online sources and help to manage expectations with regards to perceived benefits. An integrated approach, which periodically identifies patients’ needs throughout the cancer journey can better inform HCPs, who will then be able to make safe, evidence-based recommendations.^53^ The influence of misinformation is seen across the entirety of the cancer journey, with some patients with treatable disease opting out of evidence-based regimens for alternative treatment approaches, or those with advanced cancer and limited treatment options attempting to prolong life by exhausting all possibilities.^40^ Although BBCAM users tend to be well educated, having a high level of education does not necessarily preclude an individuals’ struggle to understand the complex medical vocabulary found in health-related material.^41^ Increasing access to oncology-specific health and social care professionals such as dietitians, psychologists and physiotherapists may help to address needs. It is essential that these services be available throughout the cancer journey, which is life-long and does not end on completion of conventional treatment.

## Conclusion

Despite little evidence to support safety and efficacy, the use of BBCAM increases significantly in Irish cancer survivors post cancer diagnosis. Current cancer care provision needs to be remodelled using a more integrated approach, with a greater emphasis placed on evidence-based cancer survivorship. Cancer survivors are a heterogeneous population with diverse needs, and all measures should be taken to ensure the safety of this potentially vulnerable cohort. This should include training for HCPs to engage in patient-centred discussions, encouraging open disclosure while also mandating for the regulation of CAM therapists and products. The formulation of evidence-based resource should also be a priority. Future public health policies which aim to combat cancer and BBCAM misinformation, particularly on online platforms, is especially warranted.

## Supplementary Material

oyag127_Supplementary_Data

## Data Availability

The datasets generated and/or analysed during the current study are available from the corresponding author on reasonable request.
