# Supplementary material for: Exploring biologically-based complementary and alternative medicine use among Irish cancer survivors: findings from a national survey
Source: Oncologist. 2026 Apr 29;31(6):oyag127. doi: 10.1093/oncolo/oyag127 (PMC13153693; doi:10.1093/oncolo/oyag127)
Supplement: oyag127_Supplementary_Data [file oyag127_supplementary_data.pdf]

A National Survey to Investigate the Use of Complementary & Alternative Medicine in Irish Oncology Patients (OncAMIE)

Participant Information Form v.4 (Survivor)

This study is being conducted by the Nutrition & Oncology Research Group, University College Cork and is funded by the Irish Cancer Society grant ASTA19RY.

Aim of the survey

We would like to hear from patients at every stage of their cancer journey, from diagnosis right through to those who have achieved long-term remission.

Please note that when we say ‘cancer survivors’ we are referring to people who have ever been diagnosed with cancer – you are considered a survivor if you have just been diagnosed, if you are receiving active treatment or if you have been cured or are receiving palliative care.

This survey should take approximately 45 minutes and there are 5 sections with 77 questions (although some questions will only appear if they are relevant to you).

We want to hear your opinions of complementary and alternative therapies; whether you use them and how you use them. We will also ask various questions about you and your cancer to see if these details are related to your use of complementary or alternative medicine.

We hope you will take the opportunity to have your say on this important aspect of cancer care but your participation is entirely voluntary and will not impact your cancer care in any way.

Potential Risks & Benefits

We do not foresee any serious risks or benefits for you as an individual completing this survey. However, you may be distressed by thinking about your cancer or treatment. Some questions may be uncomfortable for you, <u>but you may skip any question you do not wish to answer.</u> We will leave contact details for support organisations at the end of the survey.

While this survey is not intended to benefit you directly, you will be provided with links to reliable information about complementary or alternative medicine on completion of the survey and the results of this patient survey will help guide cancer professionals in Ireland in the future in how best to support patients who wish to use these therapies. You will also have an opportunity to receive a summary of the results when the study has finished.

Your information is kept private

Your participation in this survey is completely confidential and will not be shared with your healthcare providers. The data is recorded anonymously and cannot be linked to any one individual. As participation is voluntary, you can quit the survey at any time, however, since the survey is anonymous, it will not be possible to remove your responses once you have clicked submit.

This study has been approved by the Clinical Research Ethics Committee for the Cork Teaching Hospitals (CREC). All data collected will be stored in accordance with GDPR. Survey results will be stored securely at University College Cork for 10 years, at which point they will be destroyed.

Share your details for a follow-up interview
If you wish to participate in an additional interview, please indicate this. The contact details you provide will not be linked to your response in this survey. If you do not wish to take part in a follow-up interview, you can simply skip this section and submit your responses.

Summary of results
If you would like to receive a summary of results when this study is complete, you will have an opportunity to provide an email address at the start of the survey.

Get in touch
If you have any further questions about the study or would prefer to complete the survey by telephone with assistance, please contact Erin Sullivan (Research Dietitian) at

- 0851333050 or
- erin.sullivan@ucc.ie

If you have any further queries concerning your rights in connection with this research you should contact the Clinical Research Ethics Committee for the Cork Teaching Hospitals (CREC) at:

- Lancaster Hall, 6 Little Hanover Street, Cork
- 021 4901901 or
- crec@ucc.ie

If you wish to discuss any aspect of your cancer care, or if this survey had raised questions for you or caused any distress, please contact the Irish Cancer Society Nurseline for confidential advice, support and information on any cancer issue at:

- Freephone 1 800 200 700 or
- https://www.cancer.ie/cancer-information-and-support/cancer-support/find-support/cancer-nurseline/enquiry-form

If you are in need of confidential emotional support please contact Samaritans for free at:

- 116 123 or
- jo@samaritans.ie

If you are in severe distress and need immediate help, please contact:

- Your GP or
- Call 999 or 112 for emergency services

We would greatly appreciate if you could share this survey with anyone else you know who has had cancer. **SHORT LINK**

Please answer the questions to the best of your ability. There are no right or wrong answers.

Thank you for your participation.

Kind Regards,

Dr. Aoife Ryan RD,
Principal Investigator
CORU Registered Dietitian DI021735,
Nutrition & Oncology Research Group,
School of Food & Nutritional Sciences,
University College Cork.
a.ryan@ucc.ie

Erin Stella Sullivan RD,
Research Dietitian,
CORU Registered Dietitian DI023289,
Nutrition & Oncology Research Group,
School of Food & Nutritional Sciences,
University College Cork.
erin.sullivan@ucc.ie

A National Survey to Investigate the Use of Complementary & Alternative Medicine in Irish Oncology Patients (OncAMIE)

Informed Consent Form v.4 (Survivor)

If you consent to participating in this survey, please check the boxes to confirm the statements below and click the consent button below to continue.

A. I have read the information above and understand what is being asked of me
   a. Yes
   b. No
B. I agree to my anonymous responses being stored at University College Cork for 5 years
   a. Yes
   b. No
C. I have or have had cancer
   a. Yes
   b. No
D. I live in the Republic of Ireland
   a. Yes
   b. No
E. I am 18 years of age or older
   a. Yes
   b. No

F. Please enter full name here
G. Please enter your email address here **(G OR H required)**
H. Please enter your contact number here **(G OR H required)**

I CONSENT TO BE CONTACTED WITH AN INVITATION TO PARTICIPATE IN THE SURVEY (IF A through E = YES)

Contact Details

If you would like to take part in a further telephone interview to discuss your opinions in more detail or if you wish to receive a summary of results when the study is finished, please fill in the form below.

I. Do you consent to being contacted about a possible telephone interview?
   a. Yes
   b. No

II. Would you like to receive a summary of results when this study is finished?
   - Yes
   - No

I CONSENT TO BE CONTACTED (IF I OR II=YES)
I DO NOT CONSENT TO BE CONTACTED (IF I AND II=NO)

A National Survey to Investigate the Use of Complementary & Alternative Medicine in Irish Oncology Patients (OncAMIE)

Survey v.4 (Survivor)

Part 1: About You
The following questions ask some basic details about you

1. With which gender do you identify? *Choose the answer which best describes you*

- Male
- Female
- Other (please specify)

2. What age are you? *Answer in years*

3. What is your current marital status? *Check the option which best describes your situation*

- Single
- Married/Civil Partnership
- Unmarried Partnership/Cohabiting
- Widowed
- Separated
- Divorced
- Other (please specify)

4. What is your current employment status? *Check any that apply*

- Employed (Full-time)
- Employed Full-time (on sick leave currently)
- Employed (Part-time)
- Employed Part-time (on sick leave currently)
- Self-employed/Business Owner
- Self-employed/Business Owner (on sick leave currently)
- Homemaker
- Unemployed (since before illness)
- Unemployed (due to illness)
- Unemployed (due to COVID-19)
- Retired
- Other (please specify)

5. What is/was your occupation? *Please enter short text here*

6. What is the highest educational level which you have completed? *Check any that apply*

- Primary School
- Junior Certificate (Lower Secondary School)
- Leaving Certificate (Upper Secondary School)
- Post-Leaving Certificate Qualification (or equivalent NFQ Level 5-6)
- Ordinary University Degree (or equivalent NFQ Level 7)
- Honours University Degree (or equivalent NFQ Level 8)
- Masters/Postgraduate Diploma (or equivalent NFQ Level 9)
- Doctorate (or equivalent NFQ Level 10)
- Still in Full Time Education
- Other (please specify)
- No Formal Education

7. What is your total annual household income before tax? *Please enter value to the nearest euro*

8. Has your financial situation been negatively impacted by your cancer diagnosis? *Check the option which best describes your situation. You may add a comment in the other box if you wish*

- Yes
- No
- Other (please specify)

9. Has your financial situation been negatively impacted by COVID-19? *Check the option which best describes your situation. You may add a comment in the other box if you wish*

- Yes
- No
- Other (please specify)

10. Has your employment situation been negatively impacted by your cancer diagnosis? *Check the option which best describes your situation. You may add a comment in the other box if you wish*

- Yes
- No
- Other (please specify)

11. Has your employment situation been negatively impacted by COVID-19? *Check the option which best describes your situation. You may add a comment in the other box if you wish*

- Yes
- No
- Other (please specify)

12. What is your current living situation? *Check the option which best describes your situation*

- Own Home (No Mortgage)
- Own Home (Mortgage)
- Renting (From Local Authority or Housing Association)
- Renting (Private Tenancy)
- Other Housing (Rent Free)
- Sheltered Housing
- Homeless
- Other (please specify)

13. With what ethnicity do you identify? *Please enter short text here*

14. In which religious tradition(s) were you raised? *Please enter short text here*

15. Which religious tradition(s) do you currently practice? *Please enter short text here*

16. In which country were you born? *Please enter short text here*

17. In which country were you raised? *Please enter short text here*

18. In which Irish county do you currently live? *Please enter short text here*

19. Which of the following best describes the region in which you live? *Choose the answer which best represents your current region*

- Urban (Population more than 10,000)
- Town (Suburb)
- Town (Rural)
- Village
- Isolated Rural Dwelling
- Other (please specify)

20. How would you describe your level of English? *Check the option which best describes your level*

- Native
- Second Language (Proficient)
- Second Language (Intermediate)
- Second Language (Basic)
- No English (Survey Completed with Assistance)

21. Do you use any social media? *Check any that apply*

- Twitter
- Instagram
- Facebook
- YouTube
- Snapchat
- Pinterest
- Other (please specify)
- None of the above

22. Do you use any mainstream media? *Check any that apply*

- Radio
- Television
- Magazines
- Newspaper
- Newsletters
- Podcasts
- Other (please specify)
- None of the above

SAVE & CONTINUE

Part 2: Cancer

The following questions ask for some details about your cancer

23. Where in your body was your first cancer diagnosed? *Please enter short text here, e.g. breast, lung, pancreas*

24. What stage is/was your cancer? *Check the option which best describes your stage or if you stage is not shown here, describe in the other box*

- 0/in situ
- 1
- 2
- 3
- 4
- Other (please specify)

25. What is the current status of your disease? *Choose whichever option most closely resembles what you know about your disease at the moment*

- Active Disease
- Recurrence
- Remission/No Evidence of Disease
- Cured
- Other (please specify)

26. Has your cancer metastasised (spread to other parts of your body)? *Choose whichever option most closely resembles what you know about your disease at the moment*

- Yes
- No
- I don't know

27. Have you had a recurrence at any time? *Choose whichever option most closely resembles what you know about your disease at the moment*

- Yes
- No
- I don't know

28. How long ago were you originally diagnosed? *Provide answer in years or months*

29. Which treatments have you received (since your diagnosis)? *Check any that apply*

- Surgery
- Chemotherapy
- Radiotherapy
- Bracytherapy
- Hormone Therapy
- Immunotherapy
- Targeted Therapy
- Clinical Trial
- Other (please specify)
- None of the above

30. Which treatments are you currently receiving (or have received in the last month)? *Check any that apply*

- Surgery
- Chemotherapy
- Radiotherapy
- Bracytherapy
- Hormone Therapy
- Immunotherapy
- Targeted Therapy
- Clinical Trial
- Other (please specify)
- None of the above

31. Are you scheduled for any surgeries?

- Yes
- No
- No, but it may be required later

32. Which of the following problems are you experiencing? *Pull any that apply into the box on the left and sort these so that the problem which bothers you most is at the top, and the problem which bothers you least is at the bottom*

- Pain
- Drowsiness
- Fatigue
- Disturbed Sleep
- Nausea
- Vomiting
- Lack of Appetite
- Shortness of Breath
- Numbness/Tingling
- Weight Gain
- Weight Loss
- Body Image Concerns
- Bowel Incontinence
- Urinary Incontinence
- Anxiety
- Low Mood
- Fear of Recurrence
- Feeling Helpless
- Other Mental Health Concerns
- Sexual Health Problems
- Menopause Symptoms
- Other (please specify)
- None of the above

33. How is your current physical function? *Choose whichever option most closely resembles how you feel at the moment*

- Able to carry on normal activity and to work
- Limited but still mobile and able to work
- Unable to work - able to live at home and care for most personal needs independently
- Unable to work - some assistance needed; confined to bed or chair more than 50% of waking hours
- Unable to care for self - bed/chair-bound

SAVE & CONTINUE

<u>Part 3: Healthcare</u>
The following questions ask for some details about your access to healthcare

34. In which Irish counties are you receiving or have you received treatment for your cancer? *Please enter short text here*

35. What healthcare coverage do you have or have you had during your cancer journey? *Check any that apply*

- Drugs Payment Scheme
- GP Visit Card
- Medical Card
- Long-Term Illness Card
- Private Health Insurance
- Other (please specify)
- None of the above

36. Since being diagnosed with cancer, have you accessed any of the following services? *Check any that apply*

- Dietitian (Public)
- Dietitian (Private)
- Occupational Therapy
- Physiotherapy
- Psychologist
- Psychiatrist
- Counsellor
- Specialist Nurse
- Speech and Language Therapy
- Medical Social Work
- Other (please specify)
- None of the above

37. Has your cancer care been affected by COVID-19? *Check any that apply*

- Yes - my diagnosis was delayed
- Yes - my treatment schedule has changed
- Yes - my follow-up has been affected
- No
- I don't know

SAVE & CONTINUE

<u>Part 4: Opinions on Complementary or Alternative Medicine</u>
We are asking these questions to find out what kinds of complementary or alternative medicines you are interested in.

To make it easier to complete this section, we have written a description below of exactly what we mean by some phrases. Please read this before proceeding.

Complementary & Alternative Medicines (CAM) are a group of diverse medical and health care systems, practices, and products that are not presently considered to be part of conventional medicine.

You may be familiar with some of these and you may not be familiar with many of them. If you wish to read more about these, links will be provided at the end of the survey to online information about Complementary & Alternative Medicine.

Complementary medicine is used **together with** conventional medicine e.g. using aromatherapy to help lessen discomfort following surgery.

Alternative medicine is used **instead of** conventional medicine e.g. using a special diet to treat cancer instead of chemotherapy

Specific examples include:

Whole Medical Systems
Homeopathy, Naturopathy, Traditional Chinese Medicine, Ayurveda, Kampō, Sowa-Rigpa, Other Traditional Medical Systems.

Mind-Body Medicine
Biofeedback, Meditation, Mindfulness, Prayer, Mental Healing, Hypnotherapy, Guided Imagery, Relaxation Techniques, Creative Therapies (Art, Music, Dance Therapy).
Patient support groups and psychological interventions such as cognitive behavioural therapy are not considered complementary or alternative medicine

Biologically Based Practices
Dietary or food supplements, chelation therapy, diet therapy, herbal/botanical products, other naturally occurring products, e.g. shark cartilage/laetrile, GcMAF.
Nutritional supplements prescribed by dietitians or conventional doctors are not considered complementary or alternative medicine

Manipulative and Body-Based Practices
Chiropractic, Osteopathy, Massage, Moxibustion, Cupping, Reflexology, Scraping.
Physiotherapy and dry needling are not considered complementary or alternative medicine

Energy Medicine
Pulsed Fields, Magnetic fields, Alternating Current or Direct Current Fields, Rife Therapy (Radionics), Qi Gong, Reiki, Therapeutic Touch, Acupuncture, Acupressure, Tai Chi

38. Were you familiar with the term Complementary & Alternative Medicine, before starting this survey? *Choose whichever option most closely describes your previous knowledge*

- Yes, I understood it as described here
- Yes, but I understood it differently than described here
- No, I had never heard the term but I knew about some of the practices or products
- No, I had never heard of these practices or products
- I don't know

39. Has a health professional ever asked you about your use of complementary or alternative medicines? *Check any that apply*

- Yes (GP)
- Yes (Oncologist)
- Yes (Surgeon)
- Yes (Nurse)
- Yes (Pharmacist)
- Yes (Public Dietitian)
- Yes (Private Dietitian)
- Yes (Medical Social Worker)
- All of the above
- Other (please specify)
- None of the above

40. Have you ever discussed your interest in complementary or alternative medicines with a health professional? *Check any that apply (whether you or the healthcare professional started the conversation)*

- Yes (GP)
- Yes (Oncologist)
- Yes (Surgeon)
- Yes (Nurse)
- Yes (Pharmacist)
- Yes (Public Dietitian)
- Yes (Private Dietitian)
- Yes (Dietitian)
- Yes (Medical Social Worker)
- All of the above
- Other (please specify)
- None of the above

41. How did your healthcare professionals respond to your interest in complementary or alternative medicines? *Check any that apply (whether you or the healthcare professional started the conversation)*
SKIP IF 40 = None of the above

- Encouraged to continue
- Advised to stop
- Neither encouraged nor discouraged
- Discussed pros and cons
- They did not know about CAM
- Other (please specify)

42. Have you ever discussed your use of complementary or alternative medicines with a health professional? *Check any that apply (whether you or the healthcare professional started the conversation)*

- Yes (GP)
- Yes (Oncologist)
- Yes (Surgeon)
- Yes (Nurse)
- Yes (Pharmacist)
- Yes (Dietitian)
- Yes (Public Dietitian)
- Yes (Private Dietitian)
- Yes (Medical Social Worker)
- All of the above
- Other (please specify)
- None of the above

43. How did your healthcare professionals respond to your use of complementary or alternative medicines? *Check any that apply (whether you or the healthcare professional started the conversation)*
SKIP IF 42 = None of the above

- Encouraged to continue
- Advised to stop
- Neither encouraged nor discouraged
- Discussed pros and cons
- They did not know about CAM
- Other (please specify)

44. Would you want your healthcare professionals to discuss complementary or alternative medicines with you? *Choose whichever option most closely resembles how you feel at the moment*

- Yes
- No
- I would not mind
- Other (please specify)

45. Where do you get your information about complementary or alternative medicines? *Check any that apply*

- Internet Searches (googling a question)
- Internet Webpages (blogs, articles etc.)
- Scientific Literature
- Social Media (Facebook, Twitter etc.)
- TV
- Radio
- Podcasts
- Newspapers
- Magazines
- Books
- Friends
- Family
- Colleagues
- Acquaintances
- Other Patients
- Cancer Support Centres
- Hospital Staff
- Other (please specify)
- I don't get any information about complementary or alternative medicines

46. Where do you access complementary or alternative medicines? *Check any that apply*

- Health Food Shops
- Online Services
- Online Stores
- Private Clinic
- Private Individuals
- Other Patients
- Cancer Charities
- Hospital
- Other (please specify)
- I don't access complementary or alternative medicines

47. Do you find it easy to access trustworthy information about complementary or alternative medicine? *Choose whichever option most closely describes your opinion*

- Yes
- No
- Unsure
- Other (please specify)
- I don't look for any information about complementary or alternative medicines

48. When choosing a complementary or alternative medicine provider, have you checked any registers or used other sources to confirm qualifications or insurance to practice? *Check any that apply*

- Health and Social Care Professionals Council (CORU)
- The Complementary and Natural Healthcare Council (CNHC)
- Association of Registered Complementary Health Therapists of Ireland (ARCHTI)
- Federation of Holistic Therapists (FHT)
- British Register of Complementary Practitioners (BRCP)
- Other (please specify)
- None of the above

49. When choosing a complementary or alternative medicine provider, have you consulted any of the following to decide which provider to choose? *Check any that apply*

- LinkedIn
- Patient Testimonials
- Provider’s own website
- Facebook
- Twitter
- Other (please specify)
- None of the above

50. If you know of any sources of information about complementary or alternative medicines or providers of these products or services, please share them here. *Please enter text here*

51. Would you be interested in hearing about complementary or alternative medicine if your hospital provided routine information about it? *Choose whichever option most closely describes your opinion*

- Yes
- No
- Unsure
- Other (please specify)

52. Would you be interested in using complementary or alternative medicine if your hospital provided it? *Choose whichever option most closely describes your opinion*

- Yes
- No
- Unsure
- Other (please specify)

53. Have you ever felt pressure from any of the following to consider using complementary or alternative medicines for your cancer? *Choose whichever option most closely describes your opinion*

- Family
- Friends
- Other cancer patients
- Conventional healthcare professionals, e.g. nurses, doctors
- Social media, e.g. facebook, twitter
- Other (please specify)
- None of the above

54. To what extent are each of the following beliefs in preventing you from discussing complementary or alternative medicine with your healthcare professional? *Please rate how important these beliefs would be in limiting your willingness to have a discussionw ith your healthcare professional. If you do not believe any of the statements, you can check that box.*

	Not at all important	Not very important	Neither important nor unimportant	Somewhat important	Very important
They don’t need to know					
They would not understand					
They would disapprove					
They would not ask me directly					
Embarrassment about using complementary or alternative medicine					
Thinking you would be judged					
Fear you would be given out to					
Fear you might be refused standard care if you use complementary or alternative medicine					
Fear your usual care would be affected by the healthcare professional's knowledge of your use of complementary or alternative medicine					
It could cause tension with the healthcare professional					
It could cause an argument with the healthcare professional					
It is not relevant to mention					
It is a waste of the healthcare professionals time (not a priority)					
Your healthcare professionals do not need to know about it					
Your healthcare professionals don't know about complementary or alternative medicines					
Your healthcare professionals are biased against complementary or alternative medicines					

55. Would you tell the following healthcare professionals if you used complementary or alternative medicines? *Choose whichever option most closely describes your opinion*

	I would tell them without being asked specifically about it	I would tell them if they specifically asked about it	I would not tell them, even if they specifically asked about it
GP (Family Doctor)			
GP Practice Nurse			
Oncologist			
Surgeon			
Oncology Nurse			
Radiotherapist			
Medical Social Worker			
Dietitian			
Pharmacist			
Physiotherapist			
Occupational Therapist			
Psychologist			

56. How would you consider the safety of complementary or alternative medicines? *Choose whichever option most closely describes your opinion*

	Definitely harmless	May be harmless	Neither harmful nor harmless	May be harmful	Definitely harmful
Whole Medical Systems (e.g. Homeopathy, Naturopathy, Traditional Chinese Medicine, Ayurveda, Kampō, Sowa-Rigpa, Other Traditional Medical Systems)					
Mind-Body Medicine (e.g. Biofeedback, Meditation, Mindfulness, Prayer, Mental Healing, Hypnotherapy, Guided Imagery, Relaxation Techniques, Creative Therapies (Art, Music, Dance Therapy))					
Biologically Based Practices (e.g. Dietary or food supplements, chelation therapy, diet therapy/special diet, herbal/botanical products, other naturally occurring products, e.g. shark cartilage/laetrile, anti-neoplastons, GcMAF)					
Manipulative and Body-Based Practices (e.g. Chiropractic, Osteopathy, Massage, Moxibustion, Cupping, Reflexology, Scraping)					
Energy Medicine (e.g. Pulsed Fields, Magnetic fields, Alternating Current or Direct Current Fields, Rife Therapy (Radionics), Qi Gong, Reiki, Therapeutic Touch, Acupuncture, Acupressure, Tai Chi)					
Complementary or Alternative Medicines in general					
Complementary Medicines in general (Any of the above used alongside conventional medicine)					
Alternative Medicines in general (Any of the above used instead of conventional medicine)					

57. Do you think complementary or alternative medicines work? *Choose whichever option most closely describes your opinion*

	Definitely ineffective	May be ineffective	Neither effective nor ineffective	May be effective	Definitely effective
Whole Medical Systems (e.g. Homeopathy, Naturopathy, Traditional Chinese Medicine, Ayurveda, Kampō, Sowa-Rigpa, Other Traditional Medical Systems)					
Mind-Body Medicine (e.g. Biofeedback, Meditation, Mindfulness, Prayer, Mental Healing, Hypnotherapy, Guided Imagery, Relaxation Techniques, Creative Therapies (Art, Music, Dance Therapy))					
Biologically Based Practices (e.g. Dietary or food supplements, chelation therapy, diet therapy/special diet, herbal/botanical products, other naturally occurring products, e.g. shark cartilage/laetrile, anti-neoplastons, GcMAF)					
Manipulative and Body-Based Practices (e.g. Chiropractic, Osteopathy, Massage, Moxibustion, Cupping, Reflexology, Scraping)					
Energy Medicine (e.g. Pulsed Fields, Magnetic fields, Alternating Current or Direct Current Fields, Rife Therapy (Radionics), Qi Gong, Reiki, Therapeutic Touch, Acupuncture, Acupressure, Tai Chi)					
Complementary or Alternative Medicines in general					
Complementary Medicines in general (Any of the above used alongside conventional medicine)					
Alternative Medicines in general (Any of the above used instead of conventional medicine)					

58. How do you believe complementary or alternative medicines work? *Choose whichever option most closely describes your opinion*

	I do not think it has any effect	I think it has a placebo effect (has some effect because you believe it works)	I think it has true biological effects	I think there is a combination of true effects and placebo effects
Whole Medical Systems (e.g. Homeopathy, Naturopathy, Traditional Chinese Medicine, Ayurveda, Kampō, Sowa-Rigpa, Other Traditional Medical Systems)				
Mind-Body Medicine (e.g. Biofeedback, Meditation, Mindfulness, Prayer, Mental Healing, Hypnotherapy, Guided Imagery, Relaxation Techniques, Creative Therapies (Art, Music, Dance Therapy))				
Biologically Based Practices (e.g. Dietary or food supplements, chelation therapy, diet therapy/special diet, herbal/botanical products, other naturally occurring products, e.g. shark cartilage/laetrile, anti-neoplastons, GcMAF)				
Manipulative and Body-Based Practices (e.g. Chiropractic, Osteopathy, Massage, Moxibustion, Cupping, Reflexology, Scraping)				
Energy Medicine (e.g. Pulsed Fields, Magnetic fields, Alternating Current or Direct Current Fields, Rife Therapy (Radionics), Qi Gong, Reiki, Therapeutic Touch, Acupuncture, Acupressure, Tai Chi)				
Complementary or Alternative Medicines in general				
Complementary Medicines in general (Any of the above used alongside conventional medicine)				
Alternative Medicines in general (Any of the above used instead of conventional medicine)				

59. To what extent have the following factors impacted your use of complementary or alternative medicines? *Choose whichever option most closely describes your situation.*

	I was not impacted by this at all	This mildly limited my use	This moderately limited my use	This severely limited my use
High cost of products				
High cost of therapies				
Lack of trustworthy information				
Negative opinions of friends				
Negative opinions of family				
Negative opinions of other cancer patients				
Negative opinions of society in general				
Negative opinions of healthcare professionals				
Discouragement from healthcare professionals				
COVID-19 restrictions				
Poor local access to therapists				
Poor local access to products				
Poor online access to therapists				
Poor online access to products				

60. What positive effects do you think are possible from complementary or alternative medicine? *Check any that apply*

- Kill the tumour
- Slow tumour growth
- Optimise the effects of conventional treatment
- Lessen side effects of conventional treatment
- Lessen symptoms of cancer
- Improve overall wellbeing
- Improve quality of life
- Reduce psychological stress
- Reduce financial stress
- Improve the healthcare professional-patient relationship
- Improve social relationships for the patient
- Provide a support network for the patient
- All of the above
- Other (please specify)
- No positive effects

61. What negative effects do you think are possible from complementary or alternative medicine? *Check any that apply*

- Enhance tumour growth
- Interact with conventional treatment
- Worsen side effects of conventional treatment
- Worsen symptoms of cancer
- Cause its own side effects
- Worsen overall wellbeing
- Worsen quality of life
- Cause unnecessary psychological stress
- Cause obsessive/restrictive behaviours
- Cause financial stress
- Cause patients to delay conventional treatment
- Cause patients to refuse conventional treatment
- Cause tension in the healthcare professional-patient relationship
- Cause social problems for the patient
- All of the above
- Other (please specify)
- No negative effects

62. In your own words, why do you or do you not use complementary or alternative medicine? *Please enter text describing your thoughts on this, such as any barriers you have faced or what you expected from using complementary or alternative medicine*

SAVE & CONTINUE

<u>Part 5: Use of Complementary or Alternative Medicines</u>

We are asking these questions to determine the exact usage patterns of complementary or alternative medicines in the cancer survivor population.

63. Have you ever used complementary or alternative medicines? *Choose whichever option most closely describes your situation. Please note, the examples do not include every possible practice that falls under each category. If you use a similar practice that is not specifically listed, you should still check the box which says you use this*

	Yes, as well as conventional treatments	Yes, instead of conventional treatments	Yes, both as well as and instead of conventional treatments	No
Whole Medical Systems (e.g. Homeopathy, Naturopathy, Traditional Chinese Medicine, Ayurveda, Kampō, Sowa-Rigpa, Other Traditional Medical Systems)				
Mind-Body Medicine (e.g. Biofeedback, Meditation, Mindfulness, Prayer, Mental Healing, Hypnotherapy, Guided Imagery, Relaxation Techniques, Creative Therapies (Art, Music, Dance Therapy))				
Biologically Based Practices (e.g. Dietary or food supplements, chelation therapy, diet therapy/special diet, herbal/botanical products, other naturally occurring products, e.g. shark cartilage/laetrile, anti-neoplastons, GcMAF)				
Manipulative and Body-Based Practices (e.g. Chiropractic, Osteopathy, Massage, Moxibustion, Cupping, Reflexology, Scraping)				
Energy Medicine (e.g. Pulsed Fields, Magnetic fields, Alternating Current or Direct Current Fields, Rife Therapy (Radionics), Qi Gong, Reiki, Therapeutic Touch, Acupuncture, Acupressure, Tai Chi)				

64. Before your cancer diagnosis, how often did you use complementary or alternative medicines? *Choose whichever option most closely describes your situation*

Please note, the examples do not include every possible practice that falls under each category. If you use a similar practice that is not specifically listed, you should still check the box which says you use this

SKIP each section IF corresponding section in 63 = No

	Daily	Weekly	Monthly	Yearly	Never
Whole Medical Systems (e.g. Homeopathy, Naturopathy, Traditional Chinese Medicine, Ayurveda, Kampō, Sowa-Rigpa, Other Traditional Medical Systems)					
Mind-Body Medicine (e.g. Biofeedback, Meditation, Mindfulness, Prayer, Mental Healing, Hypnotherapy, Guided Imagery, Relaxation Techniques, Creative Therapies (Art, Music, Dance Therapy))					
Biologically Based Practices (e.g. Dietary or food supplements, chelation therapy, diet therapy/special diet, herbal/botanical products, other naturally occurring products, e.g. shark cartilage/laetrile, anti-neoplastons, GcMAF)					
Manipulative and Body-Based Practices (e.g. Chiropractic, Osteopathy, Massage, Moxibustion, Cupping, Reflexology, Scraping)					
Energy Medicine (e.g. Pulsed Fields, Magnetic fields, Alternating Current or Direct Current Fields, Rife Therapy (Radionics), Qi Gong, Reiki, Therapeutic Touch, Acupuncture, Acupressure, Tai Chi)					

65. After your cancer diagnosis/while receiving treatment, how often did you use complementary or alternative medicines? *Choose whichever option most closely describes your situation*
Please note, the examples do not include every possible practice that falls under each category. If you use a similar practice that is not specifically listed, you should still check the box which says you use this
SKIP each section IF corresponding section in 63 = No

	Daily	Weekly	Monthly	Yearly	Never
Whole Medical Systems (e.g. Homeopathy, Naturopathy, Traditional Chinese Medicine, Ayurveda, Kampō, Sowa-Rigpa, Other Traditional Medical Systems)					
Mind-Body Medicine (e.g. Biofeedback, Meditation, Mindfulness, Prayer, Mental Healing, Hypnotherapy, Guided Imagery, Relaxation Techniques, Creative Therapies (Art, Music, Dance Therapy))					
Biologically Based Practices (e.g. Dietary or food supplements, chelation therapy, diet therapy/special diet, herbal/botanical products, other naturally occurring products, e.g. shark cartilage/laetrile, anti-neoplastons, GcMAF)					
Manipulative and Body-Based Practices (e.g. Chiropractic, Osteopathy, Massage, Moxibustion, Cupping, Reflexology, Scraping)					
Energy Medicine (e.g. Pulsed Fields, Magnetic fields, Alternating Current or Direct Current Fields, Rife Therapy (Radionics), Qi Gong, Reiki, Therapeutic Touch, Acupuncture, Acupressure, Tai Chi)					

66. When you finished your active treatment, how often did you use complementary or alternative medicines? *Choose whichever option most closely describes your situation*
Please note, the examples do not include every possible practice that falls under each category. If you use a similar practice that is not specifically listed, you should still check the box which says you use this
SKIP each section IF corresponding section in 63 = No

	Daily	Weekly	Monthly	Yearly	Never	I have not finished treatment
Whole Medical Systems (e.g. Homeopathy, Naturopathy, Traditional Chinese Medicine, Ayurveda, Kampō, Sowa-Rigpa, Other Traditional Medical Systems)						
Mind-Body Medicine (e.g. Biofeedback, Meditation, Mindfulness, Prayer, Mental Healing, Hypnotherapy, Guided Imagery, Relaxation Techniques, Creative Therapies (Art, Music, Dance Therapy))						
Biologically Based Practices (e.g. Dietary or food supplements, chelation therapy, diet therapy/special diet, herbal/botanical products, other naturally occurring products, e.g. shark cartilage/laetrile, anti-neoplastons, GcMAF)						
Manipulative and Body-Based Practices (e.g. Chiropractic, Osteopathy, Massage, Moxibustion, Cupping, Reflexology, Scraping)						
Energy Medicine (e.g. Pulsed Fields, Magnetic fields, Alternating Current or Direct Current Fields, Rife Therapy (Radionics), Qi Gong, Reiki, Therapeutic Touch, Acupuncture, Acupressure, Tai Chi)						

67. At the moment, how often do you use complementary or alternative medicines? *Choose whichever option most closely describes your situation*

Please note, the examples do not include every possible practice that falls under each category. If you use a similar practice that is not specifically listed, you should still check the box which says you use this

SKIP each section IF corresponding section in 63 = No

	Daily	Weekly	Monthly	Yearly	Never
Whole Medical Systems (e.g. Homeopathy, Naturopathy, Traditional Chinese Medicine, Ayurveda, Kampō, Sowa-Rigpa, Other Traditional Medical Systems)					
Mind-Body Medicine (e.g. Biofeedback, Meditation, Mindfulness, Prayer, Mental Healing, Hypnotherapy, Guided Imagery, Relaxation Techniques, Creative Therapies (Art, Music, Dance Therapy))					
Biologically Based Practices (e.g. Dietary or food supplements, chelation therapy, diet therapy/special diet, herbal/botanical products, other naturally occurring products, e.g. shark cartilage/laetrile, anti-neoplastons, GcMAF)					
Manipulative and Body-Based Practices (e.g. Chiropractic, Osteopathy, Massage, Moxibustion, Cupping, Reflexology, Scraping)					
Energy Medicine (e.g. Pulsed Fields, Magnetic fields, Alternating Current or Direct Current Fields, Rife Therapy (Radionics), Qi Gong, Reiki, Therapeutic Touch, Acupuncture, Acupressure, Tai Chi)					

68. Has COVID-19 impacted your use of any complementary or alternative medicines? *Choose whichever option most closely describes your situation.*
Please note, the examples do not include every possible practice that falls under each category. If you use a similar practice that is not specifically listed, you should still check the box which says you use this

	I can no longer access this because of COVID-19	I have less access to this because of COVID-19	I use this more because of COVID-19	COVID-19 has not impacted my use of this
Whole Medical Systems (e.g. Homeopathy, Naturopathy, Traditional Chinese Medicine, Ayurveda, Kampō, Sowa-Rigpa, Other Traditional Medical Systems)				
Mind-Body Medicine (e.g. Biofeedback, Meditation, Mindfulness, Prayer, Mental Healing, Hypnotherapy, Guided Imagery, Relaxation Techniques, Creative Therapies (Art, Music, Dance Therapy))				
Biologically Based Practices (e.g. Dietary or food supplements, chelation therapy, diet therapy/special diet, herbal/botanical products, other naturally occurring products, e.g. shark cartilage/laetrile, anti-neoplastons, GcMAF)				
Manipulative and Body-Based Practices (e.g. Chiropractic, Osteopathy, Massage, Moxibustion, Cupping, Reflexology, Scraping)				
Energy Medicine (e.g. Pulsed Fields, Magnetic fields, Alternating Current or Direct Current Fields, Rife Therapy (Radionics), Qi Gong, Reiki, Therapeutic Touch, Acupuncture, Acupressure, Tai Chi)				

69. Which of the following Whole Medical Systems have you used? *Check any that apply*
SKIP IF 63a = No

- Homeopathic Medicine/Homeopathy
- Naturopathic Medicine/Naturopathy
- Traditional Chinese Medicine
- Ayurveda (Indian Medicine)
- Kampō (Japanese Traditional Medicine)
- Sowa-Rigpa (Tibetan Medicine)
- Other (please specify)

70. Which of the following Mind-Body Medicines have you used? *Check any that apply*
SKIP IF 63b = No

- Biofeedback
- Meditation
- Mindfulness
- Prayer
- Mental Healing
- Hypnotherapy
- Guided Imagery
- Relaxation Techniques
- Creative Therapies
- Art Therapy
- Music Therapy
- Dance Therapy
- Movement Therapy
- Yoga
- Spiritual Healing
- Aromatherapy
- Other (please specify)

71. Which of the following types of Biologically-Based Practices have you used? *Check any that apply*
SKIP IF 63c = No

- Dietary/Food Supplements, e.g. Garlic, Ginger, Turmeric/Curcumin, Coenzyme Q10
- Vitamin/Mineral Supplements
- Vitamin/Mineral IV Infusions
- Chelation Therapy
- Diet Therapy/Special Diet, e.g. Vegan, Ketogenic Diet, Kelley/Gonzalez Regimen, Gerson Therapy, Macrobiotic Diet, Bristol Diet
- Herbal Remedies/Botanicals, e.g. Mistletoe, St. John's Wort, Echinacea, Ginseng, Ginkgo Biloba, Elderberry, Valerian, Chamomile
- Natural Products, e.g. Shark Cartilage, Laetrile/Vitamin B17/Apricot Kernels
- Biological Medicines, e.g. GcMAF, Antineoplastons, 714X, Immunoaugmentative therapy, Melatonin
- Other (please specify)

72. Which of the following specific complementary or alternative medicines have you used? *Check any that apply*
SKIP IF 63c = No

- None
- 714-X/Trimethylbicyclonitramineoheptane Chloride
- Antineoplaston Therapy
- Apitherapy/Honey Therapy/Bee Venom Therapy
- Cancer Cell Treatment/CC Formula
- Cancell/Protocel/Sheridan's Formula/Jim's Juice/Crocinic Acid/JS–114/JS–101/126–F/Entelev
- Caesium Chloride/High pH Therapy
- Chelation Therapy
- Cytokine Therapy/Klehr's Autologous Tumor Therapy
- Colloidal Silver
- Coral Calcium
- Coffee Enema
- Cannabis/CBD/THC
- Black Salve
- Berberine
- Mistletoe
- Quercetin
- DHEA/Dehydroepiandrosterone
- Di Bella Therapy
- Dimethyl Sulfoxide/DMSO
- Emu Oil
- Gc-MAF/Gc Protein-Derived Macrophage Activating Factor
- Germanium
- Hydrazine Sulfate/Rocket Fuel Treatment
- Hyperbaric Chamber/Hyperbaric Oxygen Therapy (HBOT)
- Insulin Potentiation Therapy
- Krebiozen/Carcalon/Creatine/Substance X/Drug X
- Ketogenic Diet
- Intermittent Fasting
- Low Carb-High Fat
- Alkaline Diet
- Dairy Free Diet
- Green Juicing
- Detox Diet
- Gluten Free Diet (if not coeliac)
- Activated Charcoal
- Other Detox Product
- Lipoic Acid
- Miracle Mineral Supplement/MMS
- Orthomolecular Medicine/Megavitamin Therapy
- Oxygen Therapy

- Ozone Therapy
- Myers’ Cocktail
- Pangamic Acid/Vitamin B15
- Phosphorylethanolamine
- Poly-MVA
- Pregnenolone
- Protandim
- Quercetin
- Revici's Guided Chemotherapy
- RIGVIR/Virotherapy
- Shark Cartilage
- Sodium Bicarbonate/Baking Soda
- Urine Therapy/Urotherapy
- Vitacor
- IV Vitamin C/Ascorbic Acid
- Vitamin B17/Laetrile/Amygdalin/Apricot Kernels
- Other (please specify)

73. Which of the following Manipulative and Body-Based Practices have you used?
    Check any that apply
    SKIP IF 63d = No

- Chiropractic
- Osteopathic Medicine/Osteopathy
- Massage
- Moxibustion
- Cupping
- Reflexology
- Scraping
- Applied Kinesiology
- Craniosacral Therapy/CST
- Colon Cleansing/Colonic Irrigation
- Ear Candling
- Psychic Surgery
- Shiatsu
- Other (please specify)

74. Which of the following Energy Medicines have you used? *Check any that apply*
SKIP IF 63e = No

- Qi Gong
- Reiki
- Therapeutic Touch
- Acupuncture
- Acupressure
- Tai Chi
- Pulsed Fields
- Magnetic fields
- AC/DC Fields
- Rife Therapy (Radionics)
- Other (please specify)

75. How much money do you typically spend on complementary or alternative medicine per month? *Please provide your answer to the nearest euro.*

76. How have you funded your use of complementary or alternative medicine? *Check any that apply*

- Regular income
- Savings
- Loan
- Help from family or friends
- Crowdfunding
- Free services/products
- Other (please specify)

77. If you have any other comments, please write them here.

You may wish to share if you have a specific set of complementary/alternative medicines that you use or sources you consult for information about these.

Please remember to click submit after this question to save your responses. Thank you.

SAVE & SUBMIT

Thank you for taking part in our survey. Your responses have been saved.

If you wish to share this survey with family/friends/colleagues who are also on a cancer journey, you may share this link. **SHORT LINK**

If you wish to share the healthcare professional survey, you may share this link. **SHORT LINK**

If you wish to contact me, please do not hesitate to do so at:

- 0851333050 or
- erin.sullivan@ucc.ie

More information about complementary or alternative medicine

If you wish to read more about complemenary or alternative medicine, you may find the following links helpful.

Irish Cancer Society: https://www.cancer.ie/cancer-information-and-support/cancer-support/coping-with-cancer/cancer-and-complementary-therapies#:~:text=Does%20the%20Irish%20Cancer%20Society,scientific%20proof%20that%20they%20work

Macmillan: https://www.macmillan.org.uk/cancer-information-and-support/treatment/coping-with-treatment/complementary-therapies/about-complementary-therapies

US National Center for Complementary and Integrative Health: https://www.nccih.nih.gov/health/cancer-in-depth

Further Support

If you wish to discuss any aspect of your cancer care, or if this survey had raised questions for you or caused any distress, please contact the Irish Cancer Society Nurseline for confidential advice, support and information on any cancer issue at:

- Freephone 1 800 200 700 or
- https://www.cancer.ie/cancer-information-and-support/cancer-support/find-support/cancer-nurseline/enquiry-form

If you are in need of confidential emotional support please contact Samaritans for free at:

- 116 123 or
- jo@samaritans.ie

If you are in severe distress and need immediate help, please contact:

- Your GP or
- Call 999 or 112 for emergency services
